# Decadal shifts in Qingzang Plateau lake carbon dynamics (1970–2020): From predominant carbon sources to emerging sinks

**DOI:** 10.1016/j.ese.2024.100389

**Published:** 2024-01-07

**Authors:** Di Shen, Yu Li, Yafeng Wang, Shouliang Huo, Yong Liu, Junjie Jia, Shuoyue Wang, Kun Sun, Yang Gao

**Affiliations:** a College of Earth and Environment Science, Lanzhou University, Lanzhou, 730000, PR China; b State Key Laboratory of Tibetan Plateau Earth System, Environment and Resources (TPESER), Institute of Tibetan Plateau Research, Chinese Academy of Sciences, Beijing, 100101, PR China; c Chinese Research Academy of Environmental Sciences, Beijing, 100012, PR China; d College of Environmental Sciences and Engineering, Peking University, Beijing, 1008714, PR China; e Key Laboratory of Ecosystem Network Observation and Modeling, Institute of Geographic Sciences and Natural Resources Research, Chinese Academy of Sciences, Beijing, 100101, PR China; f College of Resources and Environment, University of Chinese Academy of Sciences, Beijing, 100049, PR China

**Keywords:** Carbon source, Carbon sink, Carbon exchange flux, Qingzang Plateau, Global climate change

## Abstract

The evasion of carbon dioxide (CO_2_) from lakes significantly influences the global carbon equilibrium. Amidst global climatic transformations, the role of Qingzang Plateau (QZP) lakes as carbon (C) sources or sinks remains a subject of debate. Furthermore, accurately quantifying their contribution to the global carbon budget presents a formidable challenge. Here, spanning half a century (1970–2020), we utilize a synthesis of literature and empirical field data to assess the CO_2_ exchange flux of QZP lakes. We find markedly higher CO_2_ exchange flux in the southeast lakes than that in the northern and western regions from 1970 to 2000. During this time, both freshwater and saltwater lakes served primarily as carbon sources. The annual CO_2_ exchange flux was estimated at 2.04 ± 0.37 Tg (Tg) C yr^−1^, mainly influenced by temperature fluctuations. The CO_2_ exchange flux patterns underwent a geographical inversion between 2000 and 2020, with increased levels in the west and decreased levels in the east. Notably, CO_2_ emissions from freshwater lakes diminished, and certain saltwater lakes in the QTP transitioned from carbon sources to sinks. From 2000 to 2020, the annual CO_2_ exchange flux from QZP lakes is estimated at 1.34 ± 0.50 Tg C yr^−1^, with solar radiation playing a more pronounced role in carbon emissions. Cumulatively, over the past five decades, QZP lakes have generally functioned as carbon sources. Nevertheless, the total annual CO_2_ emissions have declined since the year 2000, indicating a potential shift trend from being a carbon source to a sink, mirroring broader patterns of global climate change. These findings not only augment our understanding of the carbon cycle in plateau aquatic systems but also provide crucial data for refining China's carbon budget.

## Highlights

• The shifting of carbon sink and source status occurred on the Qingzang Plateau (QZP) lakes around 2000.
• After 2000, the annual carbon dioxide (CO_2_) flux on the QZP lakes decreased by about 0.7 Tg C yr^−1^.
• In the past half century, CO_2_ flux from lakes were mainly regulated by temperature and solar radiation.

## Graphical abstract

**Figure:**
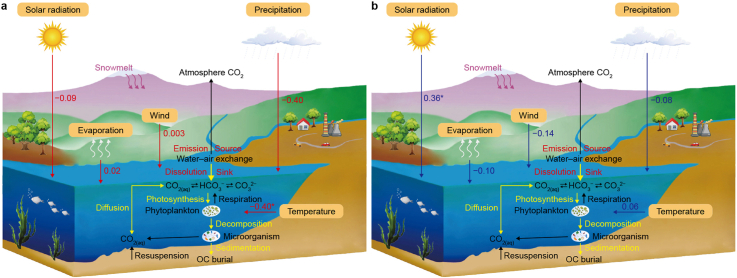

## 1 Introduction

Inland water is vital to the global carbon (C) cycle. Following decomposition and mineralization processes, an enormous amount of C will be stored in inland waters [1]. When the partial pressure of carbon dioxide (*p*CO_2_) is higher than air, carbon dioxide (CO_2_) will be released into the atmosphere through molecular diffusion and convection at the water–gas interface. Under such conditions, waterbodies act like “C sources” [2]. Globally, inland waterbodies release approximately 2.1 Pg C yr^−1^ into the atmosphere [3,4], roughly equivalent to the net C absorption rate of terrestrial ecosystems (2.6 Pg C yr^−1^) that was estimated by the Intergovernmental Panel on Climate Change (IPCC) as well as the marine C absorption rate (2.3 Pg C yr^−1^) [5]. Therefore, inland water is an important component of the global C cycle, transforming large quantities of naturally and anthropogenically derived C [6]. Therefore, even though lakes only account for approximately 3.7% of the global land area [7,8], CO_2_ emissions from the water–air interface of lakes account for approximately 15% of all emissions from inland waterbodies, thus playing an important role in regulating regional climate characteristics as well as maintaining regional carbon budgets and affiliated ecosystem balance.

CO_2_ concentrations in waterbodies are jointly affected by biophysical and chemical processes, such as carbonate thermodynamic balance, photosynthesis, respiration, and decomposition [9]. In the context of global climate change, natural and anthropogenic factors will further perplex complex CO_2_ generation and emission processes, affecting CO_2_ exchange rates at the water–air interface. In recent decades, global temperatures have increased significantly, and high temperatures can affect the gas solubility of water [10], as well as the activity of microorganisms in water and the photosynthetic process of phytoplankton [11,12]. Additionally, under strengthening precipitation and an increase in river runoff in recent years [13], many land-based substances (i.e., heavy metals and terrestrially derived nutrients into waterbodies) will inhibit or promote the growth of aquatic plants, thus affecting lake C source and sink functions [14,15]. The pH level of water also has a significant impact on lake C cycles. For example, pH will directly affect the dynamic balance and distribution of the carbonate system (CO_2_, CO_3_^2−^, and HCO_3_^−^) in water and also control CO_2_ concentration. When the water body is alkaline, the free CO_2_ in the water is converted into carbonate, and *p*CO_2_ decreases accordingly, allowing the water body to absorb CO_2_ from the atmosphere [16,17]. Moreover, higher wind speeds will accelerate the gas exchange at the water–air interface [18]. Adequate dissolved oxygen in water is beneficial for phytoplankton to maintain their life activities [19], but excessive salinity may poison phytoplankton cells [20]. In the context of global climate change, a comprehensive understanding of the carbon cycle's driving forces in lake ecosystems is crucial to advance our knowledge of inland water carbon emissions and to elucidate lakes' roles as either carbon sources or sinks.

The Qingzang Plateau (QZP), the world’s highest and largest plateau area and altitude, with the largest area, highest elevation and largest number of plateau lakes in the world [21]. Additionally, it is one of the world’s most sensitive regions to global climate change [22,23]. In recent decades, the QZP has experienced a significant temperature increase, with a warming rate double the global average, alongside increases in annual precipitation, decreases in evaporation, glacier retreats, river runoff increases, and lake area expansions [24,25]. Specifically, lakes larger than 1 km^2^ have grown from 1080 in 1970 to 1415 in 2020, with a corresponding total area increase from 4.01 × 10^4^ to 5.04 × 10^4^ km^2^ (See described in section 3.1). This expansion impacts the biophysical and chemical processes within these water bodies, influencing the C cycle on the QZP and globally. Furthermore, there is no consensus on whether QZP lakes act as C sources or sinks. Researchers are divided on whether the QZP, known for its vast concentration of saltwater and alkaline lakes (about 70% of the plateau's total lake area), functions primarily as a C source or sink. For instance, Li et al. [26] reported that saltwater lakes on the QZP act like a tremendous C sink, absorbing approximately 10.28 ± 1.65 Tg C yr^−1^ from the atmosphere, nearly a third of the net terrestrial ecosystems carbon sink on the QZP. Conversely, other researchers have reported that QZP lakes act like a C source. For example, Ran et al. [6] observe an increasing trend in C emissions from these lakes, with annual emissions rising from 2.10 ± 1.00 Tg C yr^−1^ in 1980 to 3.80 ± 1.10 Tg C yr^−1^ in 2010. This discrepancy underscores the necessity for accurate lake C flux estimation on the QZP. Furthermore, since the 21st century's onset, the QZP has experienced accelerated warming and significant increases in precipitation, outpacing other global regions [27,28]. These climatic changes induce fluctuations in water temperature, influencing the carbon source and sink patterns in the lakes. Therefore, a comprehensive understanding of the CO_2_ exchange mechanisms at the water–air interface of QZP lakes is crucial. Such knowledge will enable us to determine the carbon source and sink patterns and characteristics of these lakes more precisely, contributing to a more accurate global climate change impact assessment, particularly in terms of carbon emissions.

The objectives of this study were as follows: (1) to quantify and analyze the dynamic changes of lakes on the QZP during 1970–2000 and 2000–2020; (2) to elucidate the patterns and characteristics of C sources and sinks in various types of lakes, and across different altitudes and area gradients on the QZP during 1970–2000 and 2000–2020, estimating and comparing annual CO_2_ emissions and change trends in the QZP for these intervals; (3) to explore the mechanisms influencing the changes in lake C source and sink on the QZP under a background of global climate change.

## 2 Materials and methods

### 2.1 Study area

The study area is located on the QZP (26°00′–39°47′ N, 73°19′–104° 47′ E). The total area of the QZP is approximately 2.5 × 10^6^ km^2^, with an average altitude greater than 4500 m above sea level (MASL) [29,30]. The annual average temperature of the QZP was 3.39 °C, ranging from −19.5 °C (early February) to 25.1 °C (late July) [29], which is mainly affected by the westerlies, the East Asian monsoon, the South Asian Monsoon, and other such atmospheric circulation [31]. The region's topography, particularly its mountain systems, is crucial in its climatic diversity. These mountains act as barriers to warm and humid airflows from the south, resulting in an average annual precipitation of 2000 mm in the southeast and less than 50 mm in the northwest [32]. Solar and thermal conditions are strong on the QZP, with annual sunshine hours of 2500–3200 h [33].

**Fig. 1:**
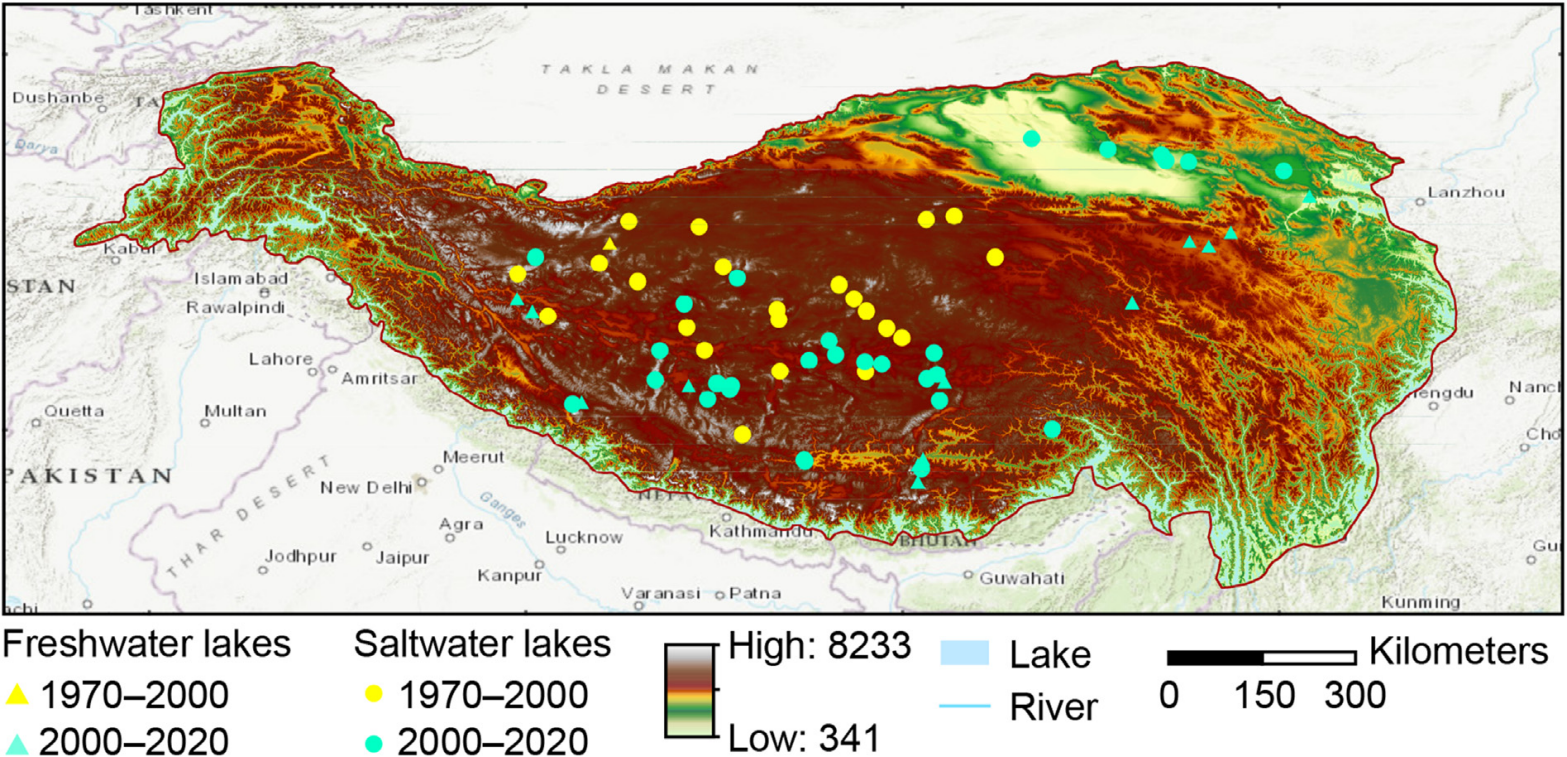
Location of the study area and sampling point distribution.

The total area of lakes larger than 1 km^2^ on the QZP spans approximately 5.04 × 10^4^ km^2^, accounting for 57% of the total lake area in China [34]. Due to complex geomorphological characteristics and climatic conditions, lakes that have formed on the QZP have the highest altitude, the widest distribution range, and the largest number of all plateau lakes in the world, including a unique concentration of saltwater and saline lakes [35]. These lakes are ideal areas to research the response of CO_2_ released by lake ecosystems to the global carbon cycle. Currently, most research has shown that the CO_2_ emissions from these lakes exceed their absorption capabilities [6] (Fig. 1).

### 2.2 Data sources

In this study, a total of 73 lakes were selected, encompassing two time periods: 29 lakes taken from 1970 to 2020 and 44 lakes from 2000 to 2020, approximately spanning a 50-year period. The area covered by the 29 lakes in the 1970–2020 period was 6232 km^2^, while the 44 lakes in the 2000–2020 period covered an area of 16,357.53 km^2^. Lake area data was derived from the Third Pole Environment Data Center [36]. Hydrochemical lake data on the QZP from 1970 to 2000 derived from the Annals of Lakes in China [37]. Data for ten lakes from 2000 to 2020 were collected from field sampling and observations, while hydrochemical data on the remaining 34 lakes were obtained from the literature (shown in Table S1). The data are all from the ice-free period. Elevation data were obtained from the Resource and Environmental Science Data Center (https://www.resdc.cn). Temperature, precipitation, evaporation, and solar radiation data were obtained from the Climate Data Store (https://cds.climate.copernicus.eu/cdsapp#!/search?type=dataset), with a spatial resolution of 1 km. Wind speed data were obtained from the QZP Data Center [38], with a data resolution of 10 km. Due to inconsistent data resolution, bilinear interpolation was used to resample meteorological data, resulting in a unified data resolution of 1 km. Atmospheric CO_2_ concentration data were obtained from the WMO Greenhouse Gas Bulletin (https://library.wmo.int/index.php?lvl=notice_display&amp;id=3030#.YqHZIdDP2Um).

### 2.3 Calculation and statistics

#### 2.3.1 *p*CO_2_

Dissolved inorganic carbon (DIC) in water is mainly composed of dissolved CO_2_, H_2_CO_3_, HCO_3_^−^, and CO_3_^2−^. The content of each component is prone to be affected by the external environment, such as water temperature and the ionic strength of the aqueous solution. According to the carbonic acid balance principle and Henry's law, lake ${p\text{CO}}_{2}$ was calculated as equations (1), (2), (3), (4) [[39], [40], [41]]:

(1) CO_2_ + H_2_O ⇌ H_2_CO_3_∗ ⇌ H^+^ + HCO_3_^−^ ⇌ 2H^+^ + CO_3_^2−^

(2) $${K_{\text{CO}}}_{2}=\frac{\left[{H_{2}{\text{CO}}_{3}}^{*}\right]}{{p\text{CO}}_{2}}$$

(3) $$K_{1}=\frac{{[H}^{+}][{{\text{HCO}}_{3}}^{-}]}{\left[{H_{2}{\text{CO}}_{3}}^{*}\right]}$$

(4) $$K_{2}=\frac{{[H}^{+}]{[{\text{CO}}_{3}}^{2-}]}{{{[\text{HCO}}_{3}}^{-}]}$$

where *K*_*i*_ is the equilibrium constant, which is calculated by equations (5), (6), (7) [42]:

(5) $${{pK}_{\text{CO}}}_{2}=-7\times 10^{-}5T^{2}+0.016T+1.11$$

(6) $${pK}_{1}=1.1\times 10^{-}4T^{2}\;-\;0.012T+6.58$$

(7) $${pK}_{2}=9\times 10^{-}5T^{2}\;-\;0.0137T+10.62$$

where ${{pK}_{\text{CO}}}_{2}$, ${pK}_{1}$, and ${pK}_{2}$ represent negative logarithms of ${K_{\text{CO}}}_{2}$, $K_{1}$, and $K_{2}$, respectively, where T represents water temperature (°C).

According to Henry’s law, ${p\text{CO}}_{2}$ was calculated using equations (8), (9), (10), (11) [43,44]:

(8) $${pCO}_{2}=\frac{\left[{H_{2}{\text{CO}}_{3}}^{*}\right]}{{K_{\text{CO}}}_{2}}=\frac{{x(H}^{+})x\left({{\text{HCO}}_{3}}^{-}\right)}{{K_{\text{CO}}}_{2}K_{1}}$$

(9) $$x\left(H^{+}\right)=10^{-\left[\text{pH}\right]}$$

(10) $$x\left({{\text{HCO}}_{3}}^{-}\right)=\left[{{\text{HCO}}_{3}}^{-}\right]\times 10^{-0.5\surd I}$$

(11) $$I=0.5\times \frac{\left[K^{+}\right]+4\left[{Ca}^{2+}\right]+\left[{Na}^{+}\right]+4\left[{Mg}^{2+}\right]+\left[{Cl}^{-}\right]+4\left[{SO}_{4}^{2-}\right]+\left[{NO}_{3}^{-}\right]+\left[{{HCO}_{3}}^{-}\right]}{1000000}$$

where ${x(H}^{+})$ and $x\left({{\text{HCO}}_{3}}^{-}\right)$ are the ionic activities of $H^{+}$ and ${{\text{HCO}}_{3}}^{-}$, respectively; I is the ionic strength.

#### 2.3.2 CO_2_ exchange flux rate

At the water-air interface, CO_2_ diffusion flux is generally affected by differences in *p*CO_2_ between the atmosphere and water, temperature, wind speed, and other environmental factors. The CO_2_ diffusion flux at the water-air interface is calculated by equation (12) [[45], [46], [47]]:

(12) $$F=\left({CO}_{2_{w}}-{CO}_{2_{g}}\right)\times k=\left({pCO}_{2_{w}}\;-\;{pCO}_{2_{g}}\;\right)\times K_{H}\times k$$

where F is the CO_2_ exchange flux (mmol m^−2^ d^−1^). If F < 0, the waterbody absorbs CO_2_; if F > 0, the waterbody releases CO_2_. ${CO}_{2_{g}}$ is the CO_2_ concentration in the air above the water surface, and ${CO}_{2_{w}}$ is the CO_2_ concentration in water. $K_{H}$ is Henry's coefficient calculated by equation (13). k is the gas exchange coefficient. ${pCO}_{2_{w}}$ is the partial pressure of CO_2_ in water. ${pCO}_{2_{g}}$ is the partial pressure of CO_2_ in air.

(13) $$\log\;\left(K_{H}\;\right)=108.3865+0.01985076T_{w}\;-\;\frac{6919.53}{T_{w}}\;-\;40.45154\;\mathrm{lg}\;T_{w}+\frac{669365}{T_{w}^{2}}$$

where $T_{w}$ represents the kelvin temperature (K).

Due to the small flow velocity of lake water bodies, similar to still water bodies, the k value is mainly driven by wind speed and can be parameterized through wind speed. *k* was calculated by equation (14) [48,49]:

(14) $$k=\left[2.07+\left(0.215\times v_{10}^{1.7}\;\right)\right]\times {\left(\frac{{SC}_{{\text{CO}}_{2}}}{600}\right)}^{x}$$

where $v_{10}$ is the wind speed at 10 m above the water surface (m s^−1^); ${SC}_{{\text{CO}}_{2}}$ is the Schmidt number of CO_2_, which is dependent on temperature; *x* is dependent on *v*_10_, if $v_{10}$ < 3.7 m s^−1^, *x* = −2/3; if $v_{10}$ > 3.7 m s^−1^, *x* = −1/2.

${SC}_{{\text{CO}}_{2}}$ was calculated by equation (15) [50]:

(15) $${SC}_{{\text{CO}}_{2}}=1911.1\;-\;118.11t+3.4527t^{2}\;-\;0.44132t^{3}$$

where t is the temperature of surface water (°C).

#### 2.3.3 Statistical analysis

According to the lake classification criteria established by Dou [51] and Wang [37], as well as other relevant literature [23], lakes are classified based on their elevation, salinity levels, and surface area size. Following altitude gradients, lake systems used in this study were subdivided into low-altitude (with an altitude <3000 m), mid-altitude (with an altitude between 3000 and 4000 m), and high-altitude lake systems (with an altitude >4000 m). For salinity gradients, lake systems were subdivided into freshwater (salinity level <1‰) and saltwater lake systems (salinity level >1‰) (‰ is the unit of salinity, and 1‰ is equal to 1 g of salt dissolved in 1 L of water). For area gradients, lake systems were subdivided into large-area (with an area >500 km^2^), mid-area (with an area between 100 and 500 km^2^), and small-area lake systems (with an area <100 km^2^).

To calculate lake area changes, the study first compared the lakes present in 1970 with those in 2000. This involves identifying lakes that existed in both years and calculating the changes in their areas. Additionally, we identified new lakes that formed and disappeared between 1970 and 2000 and calculated their areas. A similar approach was used to determine the changes in lake areas from 2000 to 2020. To assess the change in the number of lakes, the study calculated the difference in the count of lakes between 1970 and 2000 and applied the same method to evaluate changes from 2000 to 2020.

According to the division of watersheds on the QZP, the average *p*CO_2_ and flux value of each sub-watershed was calculated. These averages were then multiplied by the respective lake areas within each watershed to estimate the annual CO_2_ exchange flux of QZP lakes. Given the non-normal distribution of data, Spearman’s rank correlation analysis was used to test correlations between lake CO_2_ exchange flux and global climate change indicators.

## 3 Results

### 3.1 Dynamic changes of lakes on the Qingzang Plateau (1970–2020)

**Fig. 2:**
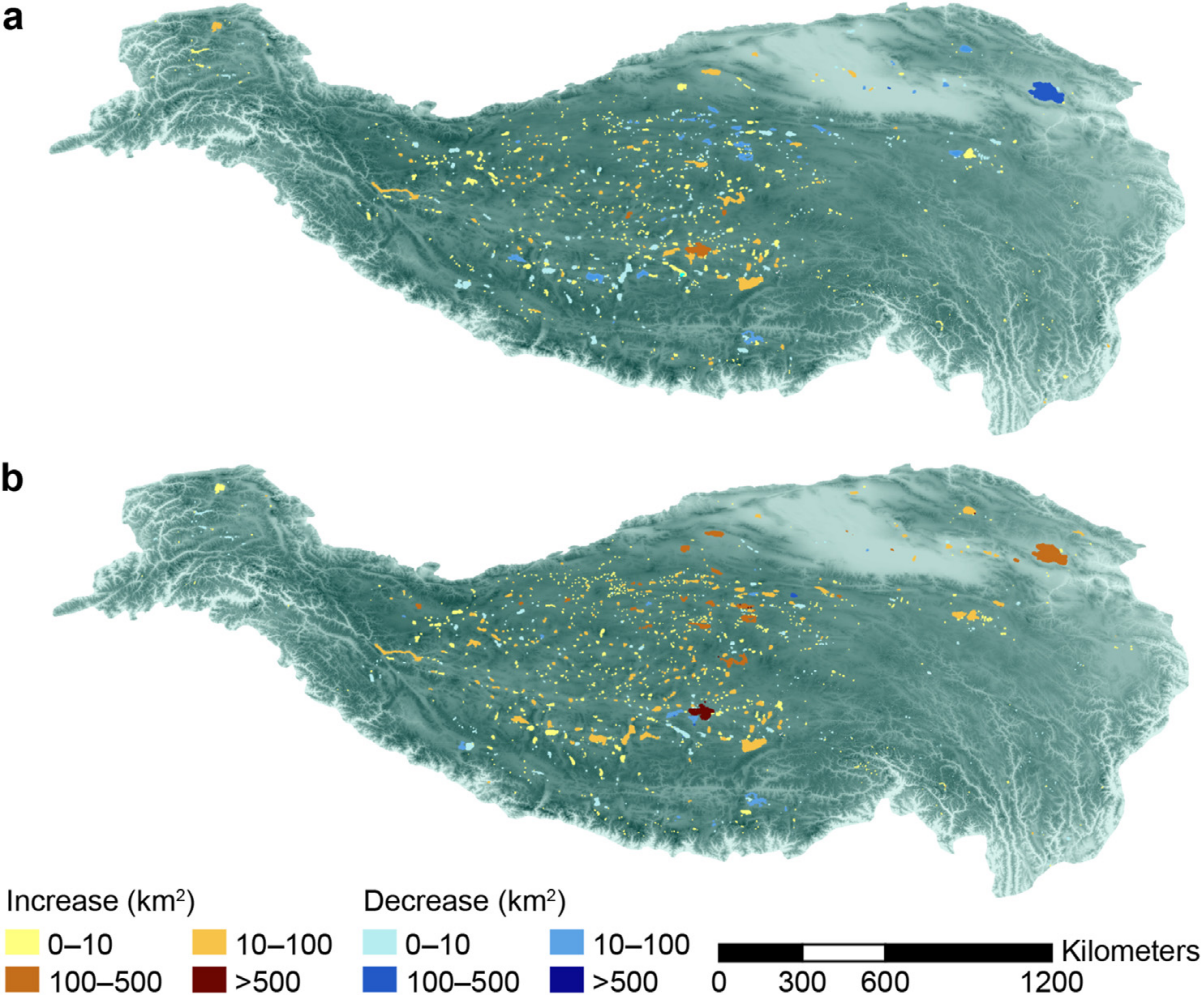
Spatiotemporal lake distribution and variation on the Qingzang Plateau during 1970–2000 (**a**) and 2000–2020 (**b**).

In 1970, 2000, and 2020, there were 1080, 1174, and 1415 QZP lakes with an area greater than 1 km^2^, with a total area of 4.01 × 10^4^, 4.09 × 10^4^, and 5.04 × 10^4^ km^2^, respectively. This data indicates a steady growth in the number and total area of QZP lakes over the past five decades. Notably, the expansion rate was relatively modest between 1970 and 2000, but it accelerated from 2000 to 2020 (Fig. 2).

**Fig. 3:**
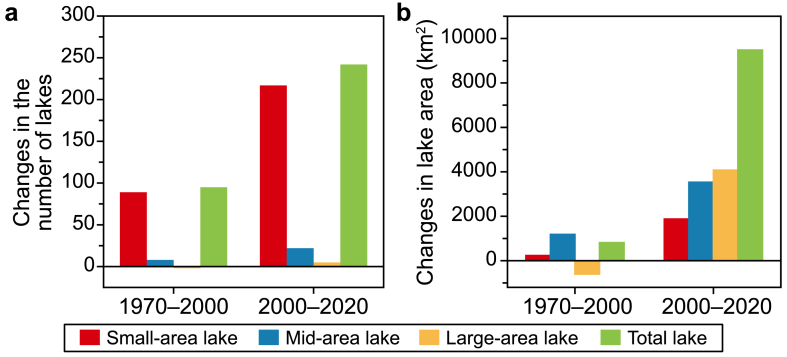
Dynamic changes in the number and area of Qingzang Plateau lakes: **a**, Changes in lake number; **b**, Changes in lake area.

From 1970 to 2000, the number of QZP lakes increased by 94, of which the number of lakes with small- and mid-area increased by 88 and 7, respectively. However, the number of large-area lakes decreased by one. The net increase in the QZP lake area is 816.23 km^2^. Moreover, mid-area lakes increased the most (i.e., an increase of 1185.45 km^2^), followed by those with small-area lakes (i.e., an increase of 232.13 km^2^). The large-area lake was reduced by 601.35 km^2^. From 2000 to 2020, there was a net increase of 241 lakes, while the number of QZP lakes with small-, mid-, and large-area increased by 216, 21, and 4, respectively. The total net increase of the QZP lake area was 9482.4 km^2^, in which the area of the large-area lake increased the most (i.e., 4076.44 km^2^), followed by the mid- and small-area lake, which increased 3531.63 and 1874.33 km^2^ respectively (Fig. 3, Table S2).

### 3.2 *p*CO_2_ characteristics

**Fig. 4:**
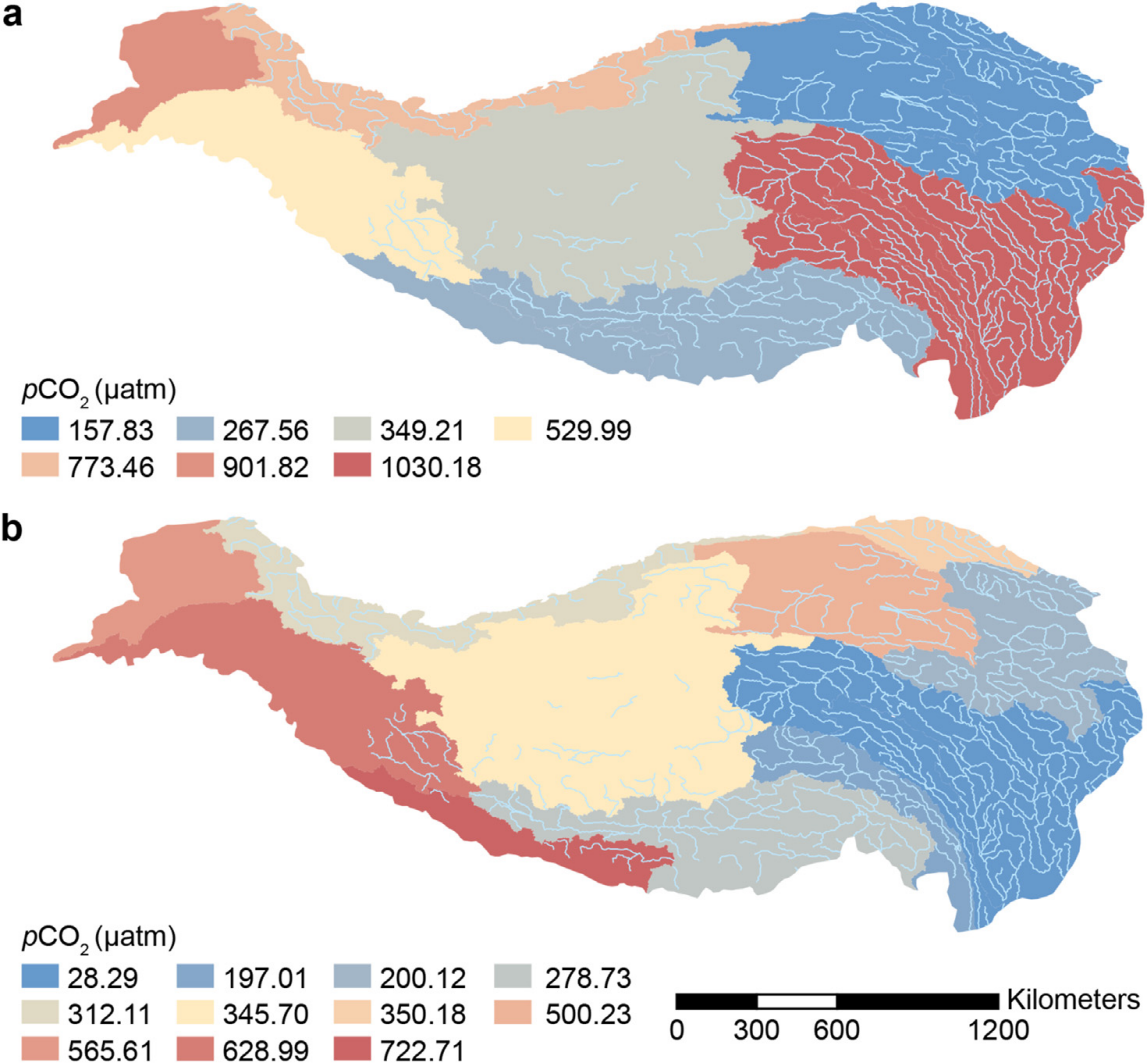
*p*CO_2_ distribution on the Qingzang Plateau lakes during 1970–2000 (**a**) and 2000–2020 (**b**).

The *p*CO_2_ of QZP lakes exhibited significant spatial differences, and freshwater lakes with large areas of high altitude were more likely to discharge CO_2_ into the atmosphere. From 1970 to 2000, the spatial distribution of *p*CO_2_ was high in the east and west and low in the north and south. The range of *p*CO_2_ in QZP lakes was between 157.83 and 1030 μatm. From 2000 to 2020, the spatial distribution pattern of *p*CO_2_ changed: high in the north, west, and southwest and low in the east and south. The range of *p*CO_2_ was between 28.29 and 722.71 μatm (Fig. 4).

**Fig. 5:**
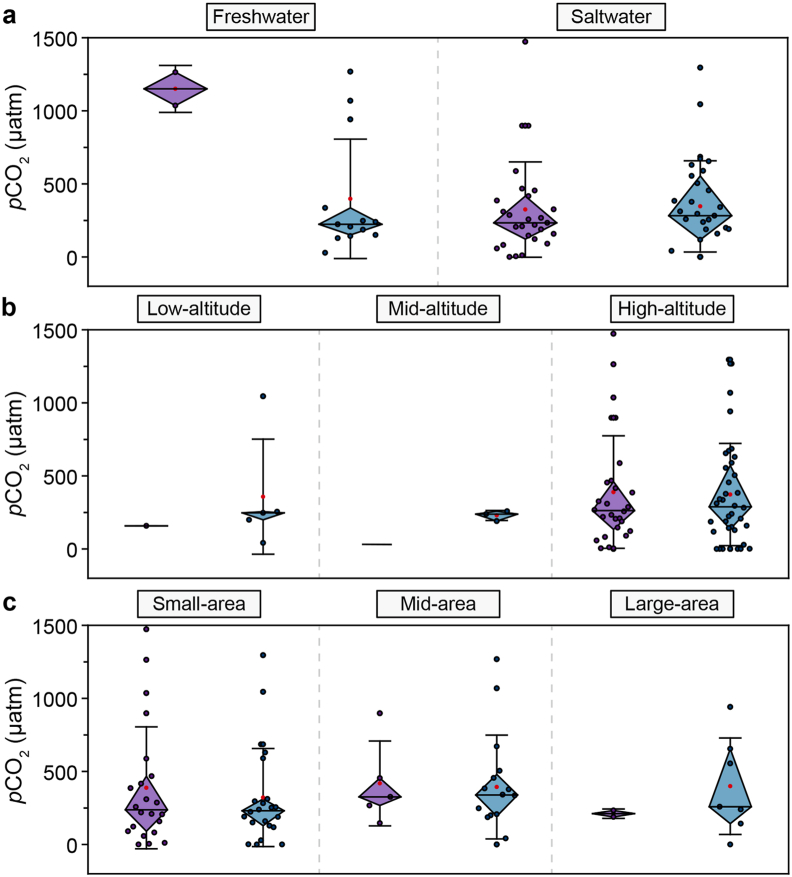
Mean *p*CO_2_ concentrations over salinity (**a**), elevation (**b**), and area (**c**) gradients (purple represents 1970–2000 and blue represents 2000–2020).

From 1970 to 2000, the *p*CO_2_ of freshwater lakes (1149.90 ± 113.53 μatm) was higher than saltwater lakes (324.42 ± 62.70 μatm). Additionally, a clear elevation-related pattern was observed: low-elevation QZP lakes had a *p*CO_2_ of 157.82 μatm, whereas high-elevation lakes exhibited a notably higher *p*CO_2_ of 389.33 ± 72.56 μatm, indicating a positive correlation between elevation and *p*CO_2_. Lake area *p*CO_2_ also exhibited certain differences. From 1970 to 2000, the *p*CO_2_ of mid-area lakes was the highest (i.e., 418.35 ± 129.80 μatm), followed by small-area lakes (388.39 ± 88.89 μatm). The small-area lakes had the lowest *p*CO_2_ value of 211.40 ± 22.56 μatm (Fig. 5). From 2000 to 2020, the *p*CO_2_ value of freshwater lakes decreased to 391.69 ± 105.00 μatm, while the *p*CO_2_ value of saltwater lakes increased to 347.30 ± 58.01 μatm. During this peroid, the *p*CO_2_ value of high-altitude lakes was higher (i.e., 372.89 ± 58.24 μatm). This phase also marked an increase in *p*CO_2_ with lake area, ranging from small (324 ± 74.90 μatm) to medium (393 ± 88.75 μatm) and large lakes (399 ± 124.66 μatm).

### 3.3 CO_2_ exchange flux

**Fig. 6:**
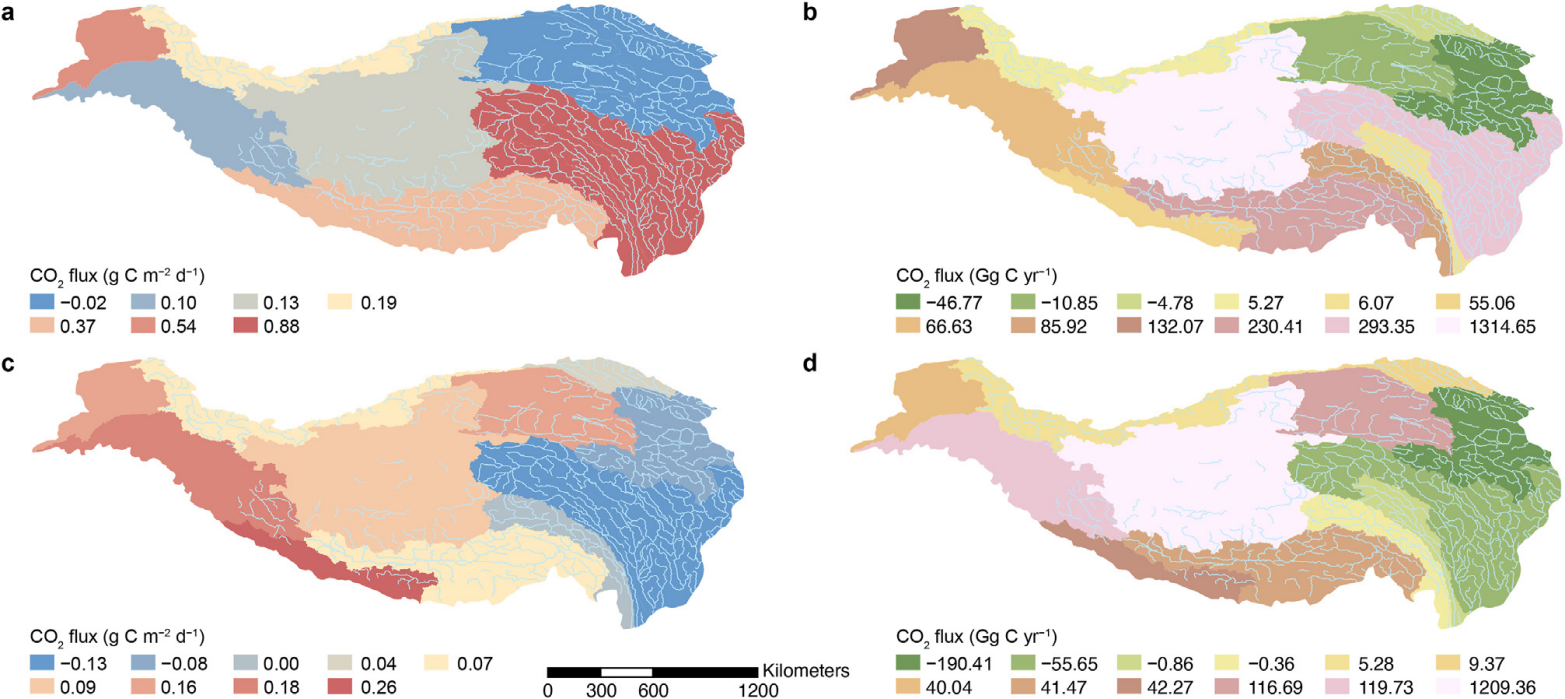
a,**c**, Distribution of lake CO_2_ flux rates on the Qingzang Plateau during 1970–2000 (**a**) and 2000–2020 (**c**). **b**,**d**, Distribution of annual CO_2_ exchange flux on the Qinghai–Tibet Plateau during 1970–2000 (**b**) and 2000–2020 (**d**).

This study revealed significant spatial differences in CO_2_ exchange flux rates and annual CO_2_ exchange flux in QZP lakes over the past five decades. From 1970 to 2000, CO_2_ exchange flux rates were high in the southeast and low in the north and west, while annual CO_2_ exchange flux were high in the central and eastern regions of the QZP and low in the north and south. During this period, the CO_2_ flux rates varied from −0.33 to 0.90 g C m^−2^ d^−1^, with an annual CO_2_ exchange flux of 2.04 ± 0.37 Tg C yr^−1^. A notable spatial shift occurred from 2000 to 2020. During this time, the CO_2_ exchange flux rates were higher in the west and lower in the east. Similarly, the annual CO_2_ exchange flux were elevated in the central and western QZP, with lower rates in the east. The CO_2_ exchange flux rate ranged from −0.23 and 0.81 g C m^−2^ d^−1^, with an annual CO_2_ exchange flux of 1.34 ± 0.50 Tg C yr^−1^. Therefore, for nearly 50 years, QZP lakes have generally acted as a C source. However, a decreasing trend was observed in annual total CO_2_ emissions from 2000 to 2020 compared to corresponding values from 1970 to 2000 (Fig. 6).

**Fig. 7:**
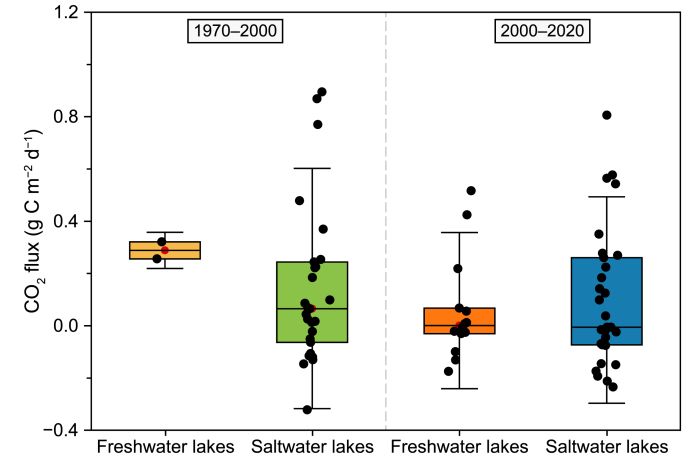
CO_2_ flux in freshwater and saltwater lakes on the Qingzang Plateau.

**Fig. 8:**
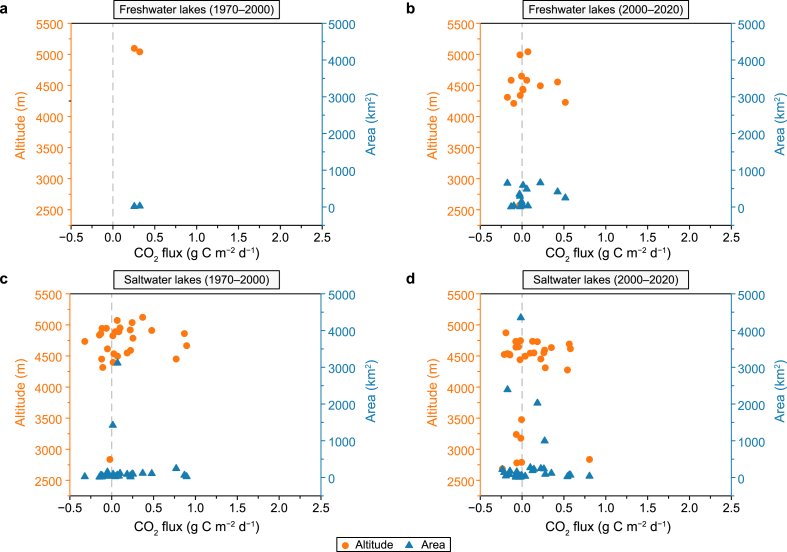
Distribution of CO_2_ flux along different altitudes and areas: **a**, Freshwater lakes from 1970 to 2000; **b**, Freshwater lakes from 2000 to 2020; **c**, Saltwater lakes from 1970 to 2000; **d**, Saltwater lakes from 2000 to 2020.

Coinciding with the turn of the 21st century, a notable shift occurred in the CO_2_ exchange flux rates between freshwater and saltwater lakes in the QZP. From 1970 to 2000, the CO_2_ exchange flux of freshwater (median = 0.29 g C m^−2^ d^−1^) and saltwater (median = 0.07 g C m^−2^ d^−1^) QZP lakes were greater than 0 and generally acted as C sources. From 2000 to 2020, CO_2_ exchange flux rates between freshwater and saltwater lakes decreased, where freshwater lakes started to act as extremely small C sources (median = 0.001 g C m^−2^ d^−1^) and the status of saltwater lakes wholly switched, shifting from C sources to C sinks (median = −0.01 g C m^−2^ d^−1^) (Fig. 7). Fig. 8 shows the CO_2_ exchange flux variation between freshwater and saltwater lakes at different elevations and area gradients. From 1970 to 2000, small- and mid-area high-altitude saltwater lakes served as C sinks, while small-area high-altitude freshwater lakes (some saltwater lakes) acted as C sources. From 2000 to 2020, some small- and mid-area high-altitude freshwater and saltwater lakes and small- and mid-area intermediate- and low-altitude saltwater lakes acted as C sinks, while small-area high-altitude saltwater lakes and small- and mid-area high-altitude freshwater lakes acted as C sources.

## 4 Discussion

### 4.1 Source and sink QZP lake characteristics

This study has revealed that QZP lake systems have generally acted as a C source during 1970–2000, with an annual CO_2_ exchange flux of 2.04 ± 0.37 Tg C yr^−1^. From 2000 to 2020, some freshwater and saltwater lakes shifted from acting as a C source to a small C sink, while the annual CO_2_ exchange flux of QZP lakes has decreased to 1.34 ± 0.50 Tg C yr^−1^. Therefore, over the past 50 years, QZP lakes have generally acted as C sources. However, since the 2000s, C emissions from QZP lakes have decreased, and the trend has shifted to a C sink.

We used a model estimation method to estimate the carbon exchange capacity at the water–air interface of lakes on the QZP. In the model estimation method, the gas exchange coefficient k is the key factor to quantify and predict the water–gas interface exchange process. In general, due to the heterogeneity of hydraulic dynamics and physical characteristics of lakes, calibration still needs to be carried out through in-situ carbon flux measurement and coefficient extrapolation. However, due to the large time scale of our research and the difficulty of sampling on the Qingzang Plateau, we cannot directly observe gas concentrations using static box methods to correct for k values. Some researchers have estimated QZP lake C emissions [6,29,52]. For example, Ran et al. [6] estimated that CO_2_ flux was 2.1 ± 1.0 Tg C yr^−1^ in the 1980s on the QZP lake. Li et al. [53] estimated that the C emissions from QZP lakes were approximately 3.60 ± 7.56 Tg C yr^−1^ in the 2010s. Jia et al. [54] estimated that the C emissions on the QZP lake were approximately 1.16 Tg C yr^−1^ in the 2020s. Our estimates of carbon emissions are consistent with these findings, falling within their reported confidence intervals. Therefore, we conclude that the carbon emissions estimates for QZP lakes derived in our study are reasonably accurate.

From 1970 to 2000, *p*CO_2_ values in QZP lakes were high and generally acted as a C source. Since the turn of the 21st century, the expansion rate in the lake area has accelerated due to increased precipitation and glacial meltwater [55,56], providing a broader living space for aquatic plants and phytoplankton growth. Additionally, due to increased river runoff, QZP lakes receive more nutrient inputs, increasing aquatic plant and phytoplankton biomass. This lake area expansion has also decreased lake salinity levels, reducing aquatic plant and phytoplankton toxicity stress [57]. Moreover, aquatic plants and phytoplankton have absorbed more CO_2_ from the atmosphere through photosynthesis to produce organic C, which has increased C fixation and decreased *p*CO_2_ values, driving some lakes to shift from a C source to a C sink. Additionally, according to World Meteorological Organization (WMO)’s Greenhouse Gas Bulletin, in 2020, atmospheric CO_2_ concentrations increased to 413.2 ± 0.2 ppm, up nearly 100 ppm from 1970’s level of 325.68 ppm [58]. This increased atmospheric CO_2_ concentrations and pressure has facilitated greater CO_2_ diffusion into aquatic systems. Consequently, these factors have collectively contributed to a decrease in QZP lake C emissions over the past five decades.

### 4.2 Mechanisms of C emission in QZP lakes

**Fig. 9:**
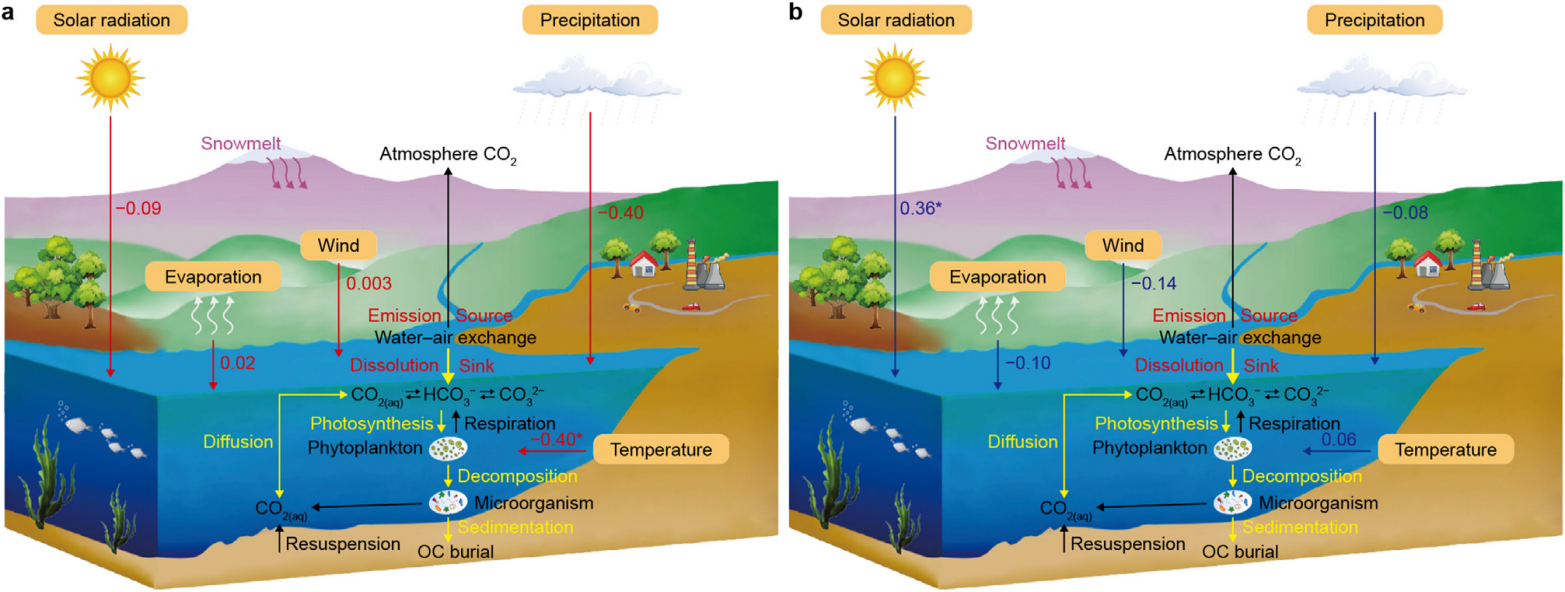
Qingzang Plateau lake C emission flux mechanisms (a represents 1970–2000 and b represents 2000–2020; red arrows represent the impacts of environmental factors on CO_2_ exchange processes from 1970 to 2000; blue arrows represent the impacts of environmental factors on CO_2_ exchange processes from 2000 to 2020; black arrows represent C decomposition and emission processes; yellow arrows represent the process of C absorption and utilization; gray arrows represent the evaporation process; purple arrows represent the flow of meltwater; numbers represent spearman’s rank correlation coefficient results between environment factors and CO_2_ flux.

At the lake water–air interface, CO_2_ exchange is typically regulated by autogenous biological activities or various environmental factors (e.g., temperature, precipitation, and wind speed) [59]. Owing to the unique environmental conditions of the QZP (i.e., high altitude, low temperatures, simple ecosystem structure, and weak anti-interference capacity), the CO_2_ exchange processes are more vulnerable to global climate change. Details on QZP lake CO_2_ exchange processes and control mechanisms are shown in Fig. 9.

From 1970 to 2000, carbon flux in QZP lakes was mainly driven by temperature, exhibiting a notable negative correlation (*P* < 0.05) (Fig. 9; Fig. S1). Temperature is another critical factor that affects and controls GHG emissions at the water–air interface. For example, temperature affects microbial activity in water and associated aquatic plant photosynthesis and respiration processes, thus impacting the rate of GHG production [54,60,61]. The annual average temperature on the QZP from 1970 to 2000 was lower than that from 2000 to 2020, while the temperature change rate before 1970 to 2000 (0.20 °C per decade) was higher than the temperature change rate from 2000 to 2020 (0.11 °C per decade). Therefore, although the temperature change rate was high from 1970 to 2000, the annual average temperature was low, aquatic plant and phytoplankton rates were relatively weak, and the effect of respiration on C emissions was more pronounced. However, after 2000, the QZP has seen an increase in annual average temperatures, and the temperature has generally had a positive effect on CO_2_. As increasing ambient temperatures have caused an increase in water temperatures on the QZP, the photosynthetic capacity of aquatic plants and phytoplankton has also been enhanced. At the same time, the CO_2_ absorbed through photosynthesis has exceeded the CO_2_ released through respiration, while *p*CO_2_ at the water-air interface has decreased, and waterbodies have increasingly become less saturated, all of which are conducive for CO_2_ entering waterbodies [10,41,62]. Therefore, in 1970–2000, temperature negatively affected CO_2_ emissions from QZP lakes, while in 2000–2020, temperature positively affected lake CO_2_ emissions. In addition, methane (CH_4_), which plays a significant role in lake carbon dynamics, is converted into carbon dioxide after oxidation into the atmosphere. Temperature has a positive effect on methane production and oxidation. Before 2000, the rate of temperature rise was fast, and methane production and oxidation rates were relatively fast. Therefore, the CO_2_ emissions from lakes on the QZP from 1970 to 2000 were higher than those from 2000 to 2020.

During 2000–2020, there was a significant positive correlation between C flux and solar radiation (*P* < 0.05) (Fig. S1; Fig. 9). Solar radiation is the main energy source of biological organisms on Earth. Smith et al. [63] found that solar radiation contributes to the photosynthesis of algal plants in lakes, reducing *p*CO_2_ in lakes. A decrease in solar radiation can lead to underwater darkening, reducing available light in the euphotic layer of lakes and impacting biological processes [64]. Especially on the QZP, most lakes are located above an altitude of 3000 m, with low levels of eutrophication. Phytoplankton and aquatic macrophytes are very sensitive to the availability of light, and a slight reduction in solar radiation will also weaken respiration processes and gradually decrease CO_2_ emissions [65,66]. In addition, solar radiation is crucial in carbon emissions during lake ice melting. Denfeld et al. [67] showed that solar radiation penetrating the ice layer is the main factor driving convection when lake ice begins to melt in spring. The circulation (flipping) of the water column caused by convection causes a significant outflow of CO_2_. In recent decades, the trend in annual solar radiation on the QZP has generally fluctuated downward. However, after 2000, the annual solar radiation on the QZP showed a trend of fluctuation and decline, which would weaken convection in the water bodies, thus reducing CO_2_ emission.

In addition to the main factors (e.g., temperature, solar radiation), other environmental factors (e.g., precipitation, evaporation, and wind speed) have also had a certain effect on the CO_2_ exchange flux in QZP lakes. In the context of global warming, changes in sea–land temperature difference between the South Asian continent and the Indian Ocean have led to the advancement and enhancement of the South Asian summer monsoon. This has resulted in stronger southwest winds, heightened water vapor convergence over the plateau, and increased precipitation [68]. Over the past 50 years, precipitation on the QZP has increased at a rate of 0.91 mm yr^−1^. Precipitation not only causes CO_2_ (and other gases) in the atmosphere to precipitate but also increases river runoff, promoting more terrestrial-based nutrients to enter waterbodies [69,70], which will increase aquatic plant and phytoplankton biomass and absorb more CO_2_ through photosynthesis. Wind shear stress will fragment surface water and increase the overall water vapor exchange area, thus increasing GHG exchange rates. With the increase in global temperature, the warming of the QZP weakens the regional meridional temperature and pressure gradients. This phenomenon has led to reduced wind speeds on the plateau, a decrease in *p*CO_2_ at the lake surface, and consequently, a reduction in CO_2_ emissions from QZP lakes to the atmosphere.

The physical and chemical properties of lakes, such as dissolved oxygen, salinity, and lake area, also significantly impact lake carbon emissions. The average elevation of the QZP exceeds 4500 MASL [71]. As altitude increases, there is a decrease in temperature and atmospheric pressure, reducing the oxygen content in water and adversely affecting aquatic organisms. Additionally, the lower atmospheric pressure at high altitudes is more conducive for gas to escape [72]. Therefore, some high-altitude lakes on the QZP exhibit C source characteristics, while most intermediate- and low-altitude lakes exhibit C sink characteristics. Salinity also affects CO_2_ exchange flux. Although phytoplankton exhibit a degree of salt tolerance, excessively high salinity levels disrupt their normal cellular osmotic pressure, hindering their normal life activities [[73], [74], [75]]. This phenomenon has led to freshwater lakes on the QZP generally functioning as carbon sinks, whereas saltwater lakes often act as carbon sources. At the same time, the number of lakes continues to expand, especially concerning the number of small- and mid-area lakes, which expand faster. From 1970 to 2020, the number of small- and mid-area lakes increased by approximately 7 per year, while the number of large lakes only increased by 4 in 50 years. Due to their extensive surface area, large lakes exhibit a certain buffering capacity; it takes longer for nutrients, metals, and other substances to diffuse throughout these larger bodies of water. Conversely, due to their limited area, small- and medium-sized lakes allow for rapid dispersion of these substances, resulting in a weaker buffering capacity. Consequently, phytoplankton in smaller lakes are more susceptible to the impact of nutrients or metals [73]. Based on these factors, after 2000, some small- and mid-area high-altitude freshwater and saltwater lakes on the QZP and small- and mid-area low- and intermediate-altitude saltwater lakes will function as C sinks.

## 5 Conclusion and perspectives

This study primarily focuses on integrating and analyzing data from various studies conducted over different periods. The significant temporal breadth of the data, spanning nearly 50 years, introduces variability in aspects such as sampling locations, times, instruments, methods, and depths. These variations, inherent to the disparate studies, potentially result in data distortions. Consequently, these inconsistencies pose challenges in accurately estimating lake carbon flux on the Qingzang Plateau, leading to certain uncertainties.

Presently, most studies estimating CO_2_ flux rely on diurnal data. However, it has been observed that *p*CO_2_ and CO_2_ flux undergo rapid daily changes. Nocturnal CO_2_ escape may also be stronger, leading to potential underestimation or overestimation of daily CO_2_ emissions based on single (one-time) measurements [76]. Most relevant studies have also reported that QZP lakes act like C sources [6,29], with these conclusions largely based on summer sampling campaigns (i.e., ice-free periods). Winter ice-cover periods, or periods of spring drift ice, have been notably underrepresented in sampling efforts, suggesting a possible bias in understanding the full C dynamics of these lakes. However, Li et al. [26] reported that saltwater lakes on the QZP can absorb approximately 10 million tons of C from the atmosphere annually, equivalent to one-third of the net productivity of the terrestrial ecosystems on the QZP. Therefore, the lack of daily lake C emission monitoring and C flux analysis data during winter ice-cover or spring drift ice periods has resulted in investigative blind spots, which could impact research results. Such omissions could lead to underestimating the C sink capacity of QZP lakes.

Over the past five decades, encompassing the years before and after the turn of the 21st century, QZP lakes have generally functioned as C sources. However, a notable shift was observed during 2000–2020, with annual CO_2_ exchange fluxes decreasing, indicating a shift trend from a C source to a C sink. This trend suggests that QZP lakes might assume an increasingly significant role in both regional and global C cycles in the context of ongoing global climate change. Therefore, in subsequent studies, field observation stations should be established to allow for continuous in situ observations of QZP lake C emissions throughout the year and to improve the temporal resolution of data to obtain more accurate C emission information. Furthermore, developing a carbon emission estimation model underpinned by remote sensing monitoring could provide a clearer answer as to whether QZP lakes are acting as carbon sources or sinks. This will provide more supportive evidence to reconstruct the ecological security patterns of alpine lakes.

## CRediT authorship contribution statement

**Di Shen:** Writing - Original Draft. **Yu Li:** Conceptualization. **Yafeng Wang:** Resources, Data Curation. **Shouliang, Huo:** Resources, Data Curation, Writing - Original Draft. **Yong Liu**: Conceptualization. **Junjie Jia:** Writing - Original Draft. **Shuoyue Wang:** Writing - Review & Editing. **Kun Sun:** Writing - Review & Editing. **Yang Gao:** Conceptualization, Resources, Data Curation, Writing - Review & Editing.

## Declaration of competing interest

The authors declare that they have no known competing financial interests or personal relationships that could have appeared to influence the work reported in this paper.
