# Supplementary material for: Decadal shifts in Qingzang Plateau lake carbon dynamics (1970–2020): From predominant carbon sources to emerging sinks
Source: Environ Sci Ecotechnol. 2024 Jan 7;21:100389. doi: 10.1016/j.ese.2024.100389 (PMC10823105; doi:10.1016/j.ese.2024.100389)
Supplement: Multimedia component 1 [file mmc1.docx]

**Table S1 Lake points on the Qinghai-Tibet Plateau**

| Number | Time | Name | Latitude (°) | Longitude (°) | Type | Altitude (m) | Area (km^2^) | The data source |
| --- | --- | --- | --- | --- | --- | --- | --- | --- |
| 1 | 1970-2000 | Yueya Lake | 34.92 | 82.22 | Freshwater | 5096 | 14.8 | [1] |
| 2 | 1970-2000 | Songmuxi Co | 34.61 | 80.25 | Freshwater | 5041 | 24.6 | [1] |
| 3 | 1970-2000 | Kyebxang Co | 32.45 | 89.98 | Saltwater | 4614 | 149 | [1] |
| 4 | 1970-2000 | Dangqiong Co | 31.57 | 86.74 | Saltwater | 4449 | 54.5 | [1] |
| 5 | 1970-2000 | Dagze Co | 31.85 | 87.51 | Saltwater | 4451 | 244.7 | [1] |
| 6 | 1970-2000 | Cedo Caka | 33.17 | 89.04 | Saltwater | 4891 | 38.5 | [1] |
| 7 | 1970-2000 | Amu Co | 33.49 | 88.70 | Saltwater | 4945 | 34.8 | [1] |
| 8 | 1970-2000 | Gangtang Co | 33.20 | 86.67 | Saltwater | 4836 | 11.3 | [1] |
| 9 | 1970-2000 | Yaggain Co | 31.56 | 89.01 | Saltwater | 4535 | 34.8 | [1] |
| 10 | 1970-2000 | Angdar Co | 32.71 | 89.58 | Saltwater | 4825 | 1422 | [1] |
| 11 | 1970-2000 | Dirangbi Co | 33.17 | 89.04 | Saltwater | 4891 | 22.1 | [1] |
| 12 | 1970-2000 | Yibug Caka | 32.94 | 86.71 | Saltwater | 4548 | 88 | [1] |
| 13 | 1970-2000 | Dong Co | 32.12 | 84.74 | Saltwater | 4396 | 87.7 | [1] |
| 14 | 1970-2000 | Rebang Co | 33.03 | 80.58 | Saltwater | 4315 | 31.6 | [1] |
| 15 | 1970-2000 | Dajia Co | 29.89 | 85.75 | Saltwater | 5122 | 114.5 | [1] |
| 16 | 1970-2000 | Yanghu Lake | 35.41 | 84.59 | Saltwater | 4786 | 90 | [1] |
| 17 | 1970-2000 | Laxiong Co | 34.34 | 85.23 | Saltwater | 4858 | 59.7 | [1] |
| 18 | 1970-2000 | Longwei Co | 33.87 | 88.31 | Saltwater | 4919 | 43.8 | [1] |
| 19 | 1970-2000 | Chabo Co | 33.36 | 84.19 | Saltwater | 4496 | 3115 | [1] |
| 20 | 1970-2000 | Angku Co | 31.18 | 85.46 | Saltwater | 4734 | 22.6 | [1] |
| 21 | 1970-2000 | Gahai | 37.13 | 97.58 | Saltwater | 2835 | 32 | [1] |
| 22 | 1970-2000 | Rena Co | 32.73 | 84.26 | Saltwater | 4589 | 17 | [1] |
| 23 | 1970-2000 | Katiao Co | 33.96 | 82.97 | Saltwater | 4944 | 16.6 | [1] |
| 24 | 1970-2000 | Chem Co | 34.16 | 79.78 | Saltwater | 4951 | 112.7 | [1] |
| 25 | 1970-2000 | Luotuo Lake | 34.44 | 81.94 | Saltwater | 5072 | 63.2 | [1] |
| 26 | 1970-2000 | Heishi North Lake | 35.56 | 82.73 | Saltwater | 5037 | 93.5 | [1] |
| 27 | 1970-2000 | Goren Co | 34.60 | 92.45 | Saltwater | 4665 | 23.5 | [1] |
| 28 | 1970-2000 | Yinma Lake | 35.60 | 90.63 | Saltwater | 4910 | 107.2 | [1] |
| 29 | 1970-2000 | Kekao Lake | 35.70 | 91.36 | Saltwater | 4860 | 62.3 | [1] |
| 30 | 2000-2020 | Nam Co | 30.79 | 90.98 | Saltwater | 4730 | 2026.00 | [2] |
| 31 | 2000-2020 | Zige Tangco | 32.05 | 90.83 | Saltwater | 4550 | 225.55 | [3] |
| 32 | 2000-2020 | Taro Co | 31.13 | 84.31 | Freshwater | 4584 | 486.62 | [3] |
| 33 | 2000-2020 | Youbu Co | 30.82 | 84.82 | Saltwater | 4617 | 63.19 | [3] |
| 34 | 2000-2020 | Dagze Co | 31.85 | 87.51 | Saltwater | 4451 | 244.70 | [3] |
| 35 | 2000-2020 | Bangong Co | 33.44 | 79.76 | Freshwater | 4494 | 658.81 | [3] |
| 36 | 2000-2020 | Songmuxi Co | 34.60 | 80.25 | Freshwater | 5042 | 29.48 | [3] |
| 37 | 2000-2020 | Cuoga Lake | 33.11 | 80.16 | Freshwater | 4339 | 6.79 | [3] |
| 38 | 2000-2020 | Langcuo Lake | 29.18 | 87.40 | Saltwater | 4747 | 8.77 | [3] |
| 39 | 2000-2020 | Keluke Lake | 37.32 | 96.87 | Saltwater | 2789 | 54.80 | [4] |
| 40 | 2000-2020 | Mapam Yumco | 30.68 | 81.47 | Freshwater | 4555 | 412.00 | [5] |
| 41 | 2000-2020 | Pagsum Co | 30.02 | 93.97 | Saltwater | 3476 | 26.98 | [6] |
| 42 | 2000-2020 | Pung Co | 31.48 | 90.90 | Saltwater | 4516 | 172.11 | [7] |
| 43 | 2000-2020 | Bangkog Co | 31.75 | 89.44 | Saltwater | 4523 | 136.34 | [7] |
| 44 | 2000-2020 | Selin Co | 31.81 | 88.99 | Saltwater | 4537 | 2391.00 | [7] |
| 45 | 2000-2020 | Serbug Co | 32.00 | 88.22 | Saltwater | 4526 | 80.90 | [7] |
| 46 | 2000-2020 | Daxiong Lake | 34.05 | 85.61 | Saltwater | 4872 | 43.46 | [7] |
| 47 | 2000-2020 | Qiduo Co | 31.24 | 85.08 | Saltwater | 4642 | 8.29 | [7] |
| 48 | 2000-2020 | Yamdrok Co | 29.18 | 90.54 | Freshwater | 4427 | 591.00 | [8] |
| 49 | 2000-2020 | Puma Yumco | 28.57 | 90.40 | Freshwater | 4992 | 293.15 | [8] |
| 50 | 2000-2020 | Kongmu Co | 29.02 | 90.40 | Freshwater | 4440 | 39.75 | [8] |
| 51 | 2000-2020 | Chen Co | 28.96 | 90.49 | Saltwater | 4440 | 42.78 | [8] |
| 52 | 2000-2020 | Qinghai Lake | 36.90 | 100.11 | Saltwater | 3177 | 4349.00 | field sampling |
| 53 | 2000-2020 | Gahai | 37.13 | 97.58 | Saltwater | 2835 | 35.80 | field sampling |
| 54 | 2000-2020 | Tuosu Lake | 37.18 | 96.98 | Saltwater | 2780 | 151.40 | field sampling |
| 55 | 2000-2020 | Xiaochaidan Lake | 37.47 | 95.45 | Saltwater | 3237 | 92.40 | field sampling |
| 56 | 2000-2020 | Xitaijinaier Lake | 37.75 | 93.42 | Saltwater | 2685 | 218.20 | field sampling |
| 57 | 2000-2020 | Shaiyin Lake | 33.33 | 96.08 | Freshwater | 4583 | 6.20 | field sampling |
| 58 | 2000-2020 | Xing Xing Hai | 34.84 | 98.12 | Freshwater | 4211 | 28.70 | field sampling |
| 59 | 2000-2020 | Ngoring Lake | 34.96 | 97.60 | Freshwater | 4310 | 646.70 | field sampling |
| 60 | 2000-2020 | Donggei Cuona Lake | 35.21 | 98.70 | Freshwater | 4228 | 247.44 | field sampling |
| 61 | 2000-2020 | Longyangxia Reservoir | 36.17 | 100.79 | Freshwater | 2571 | 348.00 | field sampling |
| 62 | 2000-2020 | Bong Co | 31.23 | 91.10 | Freshwater | 4649 | 144.00 | [9] |
| 63 | 2000-2020 | Bam Co | 31.36 | 90.64 | Saltwater | 4552 | 236.16 | [9] |
| 64 | 2000-2020 | Dawa Co | 31.25 | 85.06 | Saltwater | 4635 | 114.40 | [9] |
| 65 | 2000-2020 | Qingmuke Co | 31.24 | 85.07 | Saltwater | 4646 | 6 | [9] |
| 66 | 2000-2020 | Zhari Namco | 31.08 | 85.41 | Saltwater | 4594 | 996.90 | [9] |
| 67 | 2000-2020 | Angku Co | 31.18 | 85.45 | Saltwater | 4734 | 25.00 | [9] |
| 68 | 2000-2020 | Rinqin Xubco | 31.33 | 83.43 | Saltwater | 4736 | 188.36 | [9] |
| 69 | 2000-2020 | Angrenjin Co | 29.21 | 87.38 | Saltwater | 4275 | 24.30 | [10] |
| 70 | 2000-2020 | Lhanag Tso | 30.69 | 81.23 | Saltwater | 4545 | 268.50 | [10] |
| 71 | 2000-2020 | Cangmu Co | 32.12 | 83.55 | Saltwater | 4310 | 87.50 | [10] |
| 72 | 2000-2020 | Chabo Co | 33.36 | 84.19 | Saltwater | 4496 | 32.00 | [10] |
| 73 | 2000-2020 | Norma Tso | 32.38 | 88.04 | Saltwater | 4692 | 68.10 | [10] |

**Table S2 Changes in number and total surface area of lakes on the Tibetan Plateau**

|  | Changes of lake area from1970 to 2000 | | | | Changes of lake area from 2000 to 2020 | | | |
| --- | --- | --- | --- | --- | --- | --- | --- | --- |
| Lake class | Number of  Changed lakes | Change in number of lakes (%) | Change in total area (km^2^) | Change in  area (%) | Number of  Changed lakes | Change in number of lakes (%) | Change in total area (km^2^) | Change in  area (%) |
| Samll-area  Mid-area  Large-area  All lakes | 88  7  -1  94 | 8.70%  12.73%  -7.14%  8.70% | 232.13  1185.45  -601.35  816.23 | 1.87%  9.73%  -3.87%  2.03% | 216  21  4  241 | 19.65%  33.87%  30.77%  20.53% | 1874.33  3531.63  4076.44  9482.4 | 14.85%  26.43%  27.26%  23.16% |


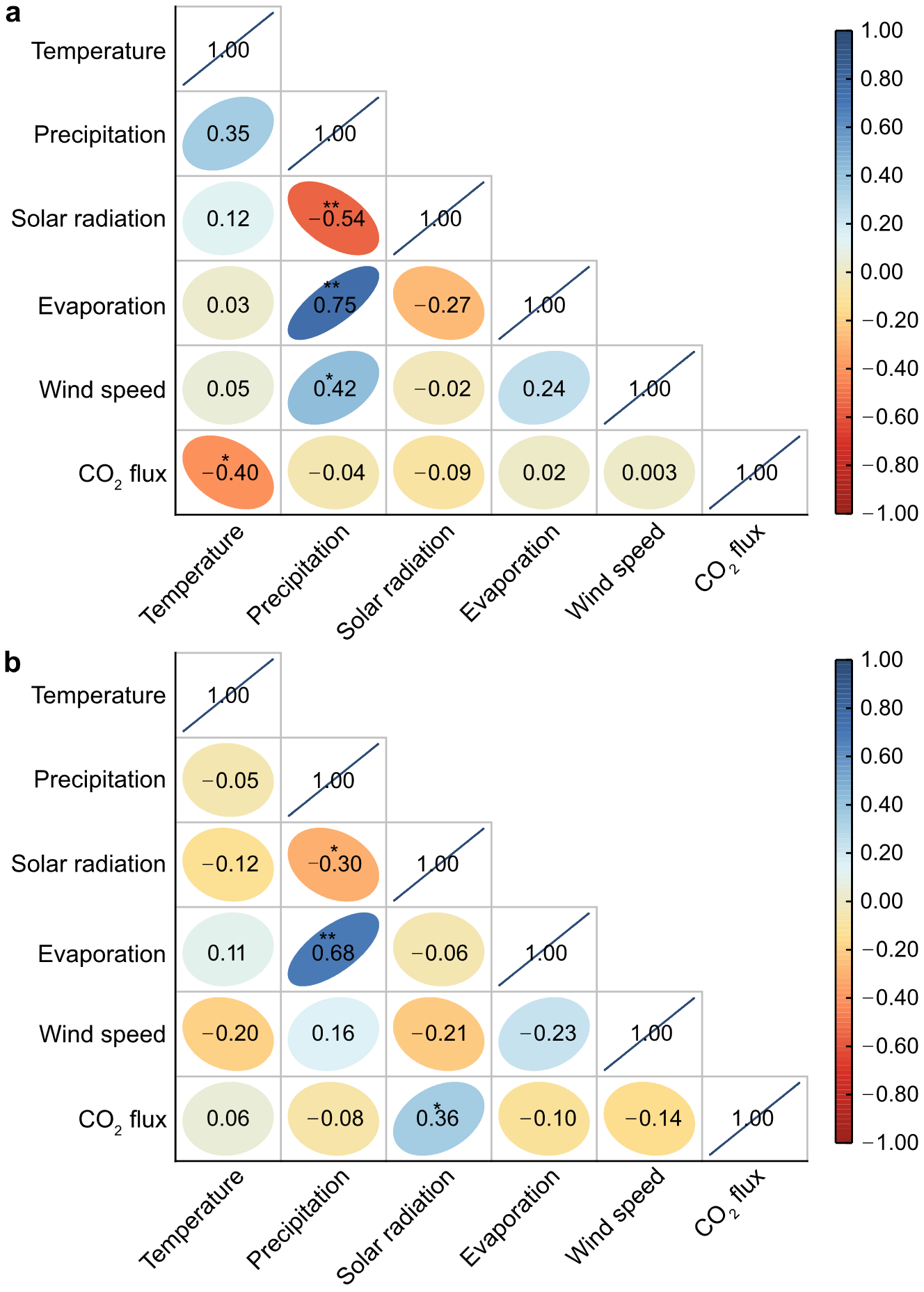


**Fig. S1.** Spearman’s rank correlation coefficient results between environment factors and CO_2_ flux (a represents 1970-2000 b represents 2000-2020).

# Reference

[1] S.M. Wang, H.S. Dou, 1998. Annals of Lakes in China (in Chinese). Beijing: Science Press.

[2] J.B. Wang, J.T. Ju, L.P. Zhu, Water chemistry variations of lake and inflowing rivers between pre- and post-monsoon season in Nam Co, Tibet (in Chinese). Scientia Geographica Sinica 33(1) (2013) 90-96.

[3] C.D. Li, S.K. Chen, Y.Q. Liu, et al., Distribution of major ions in waters and their response to regional climatic change in Tibetan lakes (in Chinese). J. Lake Sci. 28(4) (2016) 743-754.

[4] J. Li, M.H. Li, X.M. Fang, et al., Hydrochemical characteristics of the Hurleg Lake (in Chinese). Arid Land Geography 38(1) (2015) 43-51.

[5] J.B. Wang, P. Peng, Q.F. Ma, et al., Investigation of water depth, water quality and modern sedimentation rate in Mapam Yumco and La'ang Co, Tibet (in Chinese). J. Lake Sci. 25 (4) (2013) 609-616.

[6] Z. Luo, L.M. Renzeng, H.L. Chen, et al., Hydrochemical characteristics and its controlling factors of Basong Lake in cold season in Tibet (in Chinese). China Environmental Science 41(9) (2021) 4263-4270.

[7] F.P. Yan, M. Sillanpää, S.C. Kang, et al., Lakes on the Tibetan Plateau as conduits of greenhouse gases to the atmosphere. J. Geophys. Res.: Biogeosci. 123(2018) 2091–2103.

[8] X.Q. Zhang, R. Sun, L.P. Zhu, Lake water in the Yamzhog Yumco basin in South Tibetan region: quality and evaluation (in Chinese). Journal of Glaciology and Geocryology 34(4) (2012) 950-958.

[9] Y.B. Lei, T.D. Yao, Y.W. Sheng, et al., Characteristics of δ^13^C_DIC_ in lakes on the Tibetan Plateau and its implications for the carbon cycle. Hydrol. Processes 26(4) (2012) 535-543.

[10] L.X. Yan, M.P. Sun, X.J. Yao, et al., Lake water in the Tibet Plateau: Quality change and current status evaluation (in Chinese). Acta Scientiae Circumstantiae 38 (3) (2018) 900-910.
